# Association between atherosclerotic cardiovascular diseases risk and renal outcome in patients with type 2 diabetes mellitus

**DOI:** 10.1080/0886022X.2021.1893186

**Published:** 2021-03-09

**Authors:** Honghong Ren, Lijun Zhao, Yutong Zou, Yiting Wang, Junlin Zhang, Yucheng Wu, Rui Zhang, Tingli Wang, Jiali Wang, Yitao Zhu, Ruikun Guo, Huan Xu, Lin Li, Mark E. Cooper, Fang Liu

**Affiliations:** a Division of Nephrology, West China Hospital of Sichuan University, Chengdu, China; b Division of Pathology, West China Hospital of Sichuan University, Chengdu, China; c Department of Diabetes, Central Clinical School, Monash University, Melbourne, Australia

**Keywords:** Atherosclerotic cardiovascular diseases, diabetes mellitus, diabetic kidney disease, end-stage renal disease renal biopsy

## Abstract

**Aims:**

Chronic kidney disease (CKD) and diabetes mellitus increase atherosclerotic cardiovascular diseases (ASCVD) risk. However, the association between renal outcome of diabetic kidney disease (DKD) and ASCVD risk is unclear.

**Methods:**

This retrospective study enrolled 218 type 2 diabetic patients with biopsy-proven DKD, and without known cardiovascular diseases. Baseline characteristics were obtained and the 10-year ASCVD risk score was calculated using the Pooled Cohort Equation (PCE). Renal outcome was defined as progression to end-stage renal disease (ESRD). The association between ASCVD risk and renal function and outcome was analyzed with logistic regression and Cox analysis.

**Results:**

Among all patients, the median 10-year ASCVD risk score was 14.1%. The median of ASCVD risk score in CKD stage 1, 2, 3, and 4 was 10.9%, 12.3%, 16.5%, and 14.8%, respectively (*p* = 0.268). Compared with patients with lower ASCVD risk (＜14.1%), those with higher ASCVD risk had lower eGFR, higher systolic blood pressure, and more severe renal interstitial inflammation. High ASCVD risk (>14.1%) was an independent indicator of renal dysfunction in multivariable-adjusted logistic analysis (OR, 3.997; 95%CI, 1.385–11.530; *p* = 0.010), though failed to be an independent risk factor for ESRD in patients with DKD in univariate and multivariate Cox analysis.

**Conclusions:**

DKD patients even in CKD stage 1 had comparable ASCVD risk score to patients in CKD stage 2, 3, and 4. Higher ASCVD risk indicated severe renal insufficiency, while no prognostic value of ASVCD risk for renal outcome was observed, which implied macroangiopathy and microangiopathy in patients with DKD were related, but relatively independent.

## Introduction

Diabetes mellitus (DM) has become a pandemic throughout these decades. As reported by the International Diabetes Federation (IDF) in 2019, the number of people with DM was estimated to be 463 million and was thought to reach 700 million by 2045 [1], with most having type 2 diabetes mellitus (T2DM). Cardiovascular disease (CVD), one of the principal macrovascular complications of diabetes, causes 40–60% of deaths inpatients with T2DM [2]. Patients with T2DM are more likely to have higher CVD prevalence than those without T2DM. In patients who have a history of acute coronary syndrome, T2DM group has a poorer clinical outcome and higher risk of recurrent CVD [3]. As the main clinical manifestation of CVD, atherosclerotic cardiovascular disease (ASCVD) contributes greatly to the mortality of diabetic patients [4].

Diabetic kidney disease (DKD), which is clinically characterized by albuminuria and gradually developed renal dysfunction, is another proxy of microvascular complications of T2DM. DKD is a common cause of chronic kidney disease (CKD) and end-stage renal disease (ESRD) [5]. According to the 2015 annual report of China Kidney Disease Network, DKD accounts for 26.96% of CKD, and up to 27.12% among dialysis patients [6]. The association between CKD and CVD has been well studied so far. In general population, CKD is an independent risk factor for CVD and all-cause mortality [7,8], both reduced glomerular filtration rate and increased urine albumin excretion, markers of CKD, are associated with increased risk of ASCVD [9]. Furthermore, CKD patients with DM are at higher risk of reaching CVD-caused death than those without DM, despite different eGFR and albuminuria level [10]. Besides, CVD has been suggested to be an independent risk factor of kidney dysfunction and development of kidney disease in two longitudinal studies [11]. Given that extra risk of CVD mortality for CKD patients with DM is mostly brought by DKD, the possible association between DKD and CVD needs to be further explored.

Considering huge expenditure for treatment and high mortality rate of CVD, many guidelines have been formulated to instruct clinical treatment for patients developed CVD and primary prevention for those with high CVD risk. In order to identify high cardiovascular risk populations out of general people, several calculating tools are developed. The Pooled Cohort Equations (PCE), a newly developed risk calculator, contains several common clinical parameters, including age, gender, blood pressure, cholesterol profile, smoking, diabetes status and use of anti-hypertensive therapy [12]. The PCE can estimate the 10-year risk of future ASCVD event among population without existed clinical ASCVD, with well calibration and discrimination [13,14].

Although the negative prognostic impact of CKD on CVD is well established in many clinical researches, the relationship between renal outcome of biopsy-proven DKD and calculated ASCVD risk score has not been studied. In the present study, we aimed to investigate 1) the differences of clinical and pathological characteristics between those with different ASCVD risk (calculated by the PCE). 2) the association between ASCVD risk and renal outcomes of T2DM patients with biopsy-proven DKD.

## Methods

### Patients

All electronic medical records of DM patients who underwent renal biopsy and diagnosed with pure DKD in West China Hospital of Sichuan University from January 2010 to March 2018 were reviewed. General indication for renal biopsy in T2DM patients was renal damage (defined as declined eGFR and/or abnormal urinalysis) and no contraindication of renal biopsy, while absence of diabetic retinopathy (DR), obvious glomerular hematuria, overt proteinuria, rapidly decreasing eGFR and short duration of DM made renal biopsy more necessary. T2DM was retrospectively diagnosed according to established criteria by the American Diabetes Association (ADA) in 2017 [15]. Diabetic kidney disease was diagnosed and classified by at least two renal pathologists and nephrologists according to the standards of the Renal Pathology Society in 2010 [16]. Inclusion criteria were: T2DM, biopsy-proven pure DKD. Exclusion criteria were: systematic disease, coexistence with non-diabetic renal disease, eGFR < 15 mL/(min·1.73 m^2^) before renal biopsy, diagnosed CVD history, incomplete clinical data when applying PCE risk calculator. These enrolled patients were followed up for at least 1 year, and outcome-related indicators were collected at the follow-up visits. This study was approved by the ethics committee of West China Hospital of Sichuan University (approval number, 2013R01). All patients have given their inform consents.

### Clinical and pathological characteristics

Baseline clinical data were collected at the time of kidney biopsy, including age, sex, height, weight, duration of diabetes, presence of diabetic retinopathy (DR), smoking status, systolic and diastolic blood pressure, fasting blood glucose, glycosylated hemoglobin (HbA1c), serum albumin, hemoglobin, serum creatinine, eGFR (calculated by Chronic Kidney Disease-Epidemiology Collaboration formula) [17], total cholesterol, high-density lipoprotein cholesterol (HDL-C), low-density lipoprotein cholesterol (LDL-C), triglyceride and 24-h urinary protein excretion. Medication history, such as lipid-lowering agents, antidiabetic therapy and renin–angiotensin–aldosterone system inhibitor (RAASi) was collected at the time of renal biopsy as well. ASCVD was defined as fatal and non-fatal stroke, non-fatal myocardial infarction and fatal coronary heart disease [12]. The 10-year ASCVD risk was calculated by the PCE according to the American College of Cardiology and American Heart Association guideline in 2014 [18]. CKD stages were classified according to Kidney Disease: Improving Global Outcomes (KDIGO) Clinical Practice Guideline in 2017, and renal dysfunction was defined as eGFR < 60mL/(min·1.73 m^2^). Renal biopsy tissue was routinely conducted light microscopy, immunofluorescence, and electron microscopy examination to clarify renal histological change. Renal specimen was evaluated by two renal pathologists, diagnosed with DKD and classified based on the Renal Pathology Society (RPS) classification [16] (including glomerular class, interstitial fibrosis and tubular atrophy (IFTA), interstitial inflammation and arteriolar hyalinosis).

### Renal outcomes

According to patient’s individual condition, follow-up visits were performed 2–4 times per year. Results of regular renal function tests, including serum creatinine, eGFR and proteinuria levels, were collected. Renal outcomes were defined as progression to ESRD, which could be interpreted by eGFR < 15 mL/(min·1.73 m^2^) or starting renal replacement therapy (i.e. hemodialysis, peritoneal dialysis, and renal transplantation).

### Statistical analysis

Continuous variables were presented as mean ± standard deviation (SD) if according with normal distribution, or as median with interquartile range (IQR). Categorical data were described as numbers and percentages. Appropriate approach, which concluded *t* test, Mann–Whitney *U* test and χ^2^ test, was selected to compare the difference between two groups. Spearman’s correlation analysis was used to analysis the correlations between ASCVD risk and clinicopathological variables. Univariate and multivariate logistic analysis Kaplan–Meier survival curve was drawn and log-rank test was used to evaluate renal survival rate. Univariate and multivariate Cox regression were performed to analyze the association between ASCVD risk and renal outcome. Some basic clinical and pathological characteristics and parameters with *p*＜0.05 in univariate analysis were adjusted in multivariate analysis. A two-sided *p* value < 0.05 was considered statistically significant. All the data were analyzed using IBM SPSS Statistics (version 22.0).

## Results

### Clinical baseline characteristics

**Figure 1.**
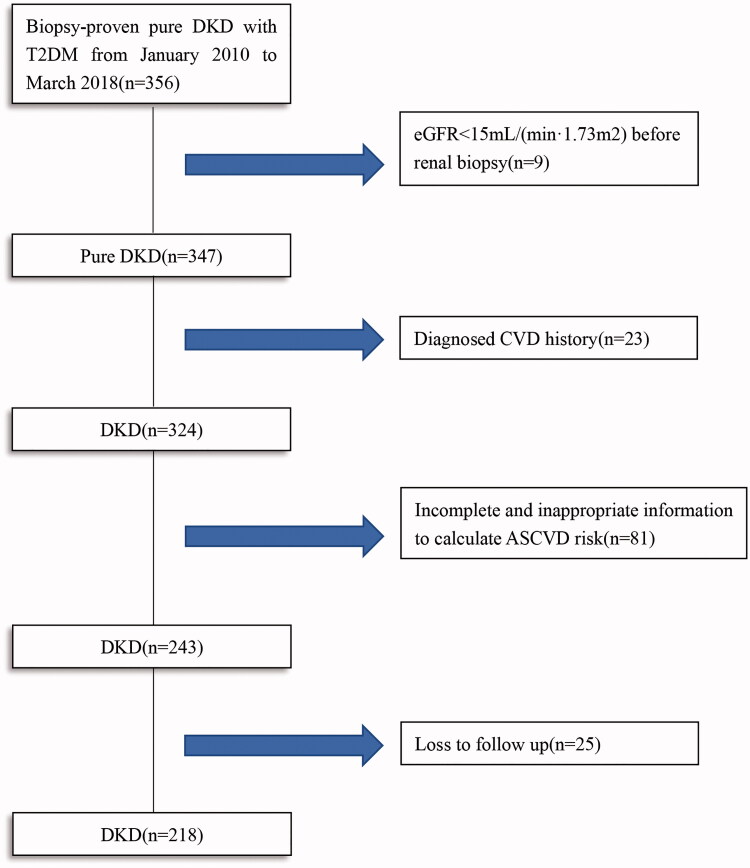
Flow chart of study participants.

In total, 356 T2DM patients with biopsy-proven pure DKD were reviewed. According to the inclusion and exclusion criteria, a total of 218 patients were enrolled in this study (Figure 1). The median follow-up time was 21 months, with 25 patients being lost follow-up. Comparation of baseline characteristics between included and excluded patients was shown in Supplementary Table 1. Of these enrolled 218 participants, the median age was 52 years with 155 (71.1%) males and 63 (28.9%) females. Ninety-six (46.7%) patients had diabetic retinopathy (DR), and smokers accounted for 48.2% (105). The median eGFR and serum creatinine was 60.75 mL/(min·1.73 m^2^) and 115.5 μmol/L, respectively.

About 75.2% patients were at high risk of 10-year ASCVD event, which was defined as 10-year ASCVD risk ≥7.5% [19]. Among group CKD1, 2, 3 and 4 stage, 27.6%, 22.2%, 28.2% and 15.4% patients had 10-year ASCVD risk ≥7.5%, respectively. Further, correlation analysis showed that ASCVD risk score was positively related to age (*r* = 0.566, *p* < 0.001), systolic blood pressure (SBP) (*r* = 0.240, *p* < 0.001) and total cholesterol (*r* = 0.176, *p* = 0.009). Males and smokers tended to have high ASCVD score (*r* = 0.495, *p* < 0.001; *r* = 0.575, *p* < 0.001) respectively. Besides, the risk score was positively associated with hemoglobin level (*r* = 0.208, *p* = 0.002), but negatively associated with baseline eGFR (*r* = –0.138, *p* = 0.043). However, no correlation between the estimated ASCVD risk score and proteinuria was found. The median ASCVD risk was 10.9% in CKD 1 stage, 12.3%in CKD stage 2, 16.5% in CKD stage 3 and 14.8% in CKD stage 4, without statistical difference (*p* = 0.268). Subgroup analyses by gender, age, smoking status, total cholesterol and HDL-C were conducted, and no significant correlation between eGFR and 10-year ASCVD risk score was observed (data not shown).

Of the total 218 patients, the median 10-year ASCVD risk was 14.1%. According to the median 10-year ASCVD risk, patients were divided into two groups: group 1 (ASCVD risk score < 14.1%; *n* = 109) and group 2 (ASCVD risk score ≥ 14.1%; *n* = 109). Compared with patients in group 1, those in group 2 had older ages (55[51–64] vs. 49[45–52] years, *p* < 0.001), male predominance (89.0% vs. 53.2%, *p* < 0.001), higher systolic blood pressure (147.71 ± 22.76 vs. 140.94 ± 20.83 mmHg, *p* = 0.023), more smokers (72.5% vs. 23.9%, *p* < 0.001), and lower HDL-C concentration (1.13[0.95–1.36] vs. 1.37[1.12–1.63] mmol/L, *p* < 0.001) and eGFR (55.14[41.11–83.54] vs. 74.28[49.52–98.06] ml/min/1.73m^2^, *p* = 0.007).There was no significant difference observed in the duration of diabetes mellitus, incidence of DR, hemoglobin, fasted blood glucose, 24-h urinary protein excretion and medical therapies between two groups (Table 1).

**Table 1.** Baseline clinical characteristics of enrolled patient.

Characteristics	All (*n* = 218)	Correlation coefficient(r)	*p* Value	ASCVD risk score groups		*p* Value
				<14.1% (*n* = 109)	≥14.1% (*n* = 109)	
Male, *n* (%)	155 (71.1)	0.495	<0.001	58 (53.2)	97 (89.0)	<0.001
Age (years)	52 (48–58)	0.566	<0.001	49 (45–52)	55 (51–64)	<0.001
DR (yes%)	99 (46.7%)	–0.056	0.419	49 (46.2%)	50 (47.2%)	0.891
BMI (kg/m^2^)	25.75 ± 4.19	0.087	0.400	25.17 ± 3.58	26.37 ± 4.72	0164
SBP (mmHg)	144.33 ± 22.03	0.240	<0.001	140.94 ± 20.83	147.71 ± 22.76	0.023
DBP (mmHg)	85.36 ± 13.01	0.074	0.274	85.19 ± 14.81	85.53 ± 10.99	0.848
Duration of diabetes (months)	96 (36–144)	0.059	0.384	96 (36–132)	108 (36–156)	0.859
Smoker, *n* (%)	105 (48.2)	0.575	<0.001	26 (23.9)	79 (72.5)	<0.001
Hemoglobin(g/L)	118.63 ± 27.62	0.208	0.002	116.05 ± 27.61	121.21 ± 27.51	0.172
FBG (mmol/L)	7.40 (5.50–9.57)	0.089	0.191	7.43 (5.41–9.56)	7.37 (5.62–9.52)	0.840
HbA1c (%)	7.30 (6.30–8.60)	0.085	0.275	7.55 (6.30–8.60)	7.30 (6.40–8.60)	0.807
eGFR (>15ml/min/1.73m^2^)	60.75 (43.31–92.50	–0.138	0.043	74.28 (49.52–98.06)	55.14 (41.11–83.54)	0.007
Serum creatinine (μmol/L)	115.5(80.0–159.0)	0.186	0.006	100.0 (72.0–144.0)	126.0 (95.2–161.0)	0.001
Uric acid (μmol/L)	384.31 ± 76.78	0.027	0.693	380.03 ± 71.47	383.58 ± 81.85	0.412
Serum albumin (g/L)	35.10 (28.70–40.20)	–0.060	0.382	35.95 (29.00–40.70)	34.50 (28.60–39.50)	0.477
Triglyceride(mmol/L)	1.78 (1.26–2.34)	0.163	0.016	1.70 (1.26–2.13)	1.86 (1.31–2.63)	0.030
Total cholesterol (mmol/L)	5.00 (4.35–5.76)	0.176	0.009	4.91 (4.26–5.60)	5.14 (4.44–5.89)	0.146
LDL-C (mmol/L)	2.90 (2.33–3.64)	0.168	0.013	2.78 (2.20–3.62)	3.03 (2.45–3.64)	0.142
HDL-C (mmol/L)	1.21 (1.02–1.53)	–0.348	<0.001	1.37 (1.12–1.63)	1.13 (0.95–1.36)	<0.001
24-h proteinuria (g/d)	4.09 (2.16–7.07)	0.125	0.079	3.59 (1.83–6.84)	4.59 (2.25–7.50)	0.127
ASCVD risk score (%)	14.1(7.7–26.8)	–	–	7.7 (4.5–10.7)	26.8 (20.0–34.0)	<0.001
Therapy						
Insulin therapy (%)	154 (70.6%)	0.033	0.625	79 (72.5%)	75 (68.8%)	0.552
Oral antidiabetic drugs (%)	99(45.4%)	–0.014	0.838	52 (47.7%)	47 (43.1%)	0.496
RAAS inhibitor (%)	175(80.3%)	0.024	0.721	85 (78.0%)	90 (82.6%)	0.395
Lipid-lowering therapy (%)	132(60.6%)	0.071	0.299	63 (57.8%)	69 (63.3%)	0.406

ASCVD: atherosclerotic cardiovascular disease; DR: diabetic retinopathy; BMI: body weight index; SBP: systolic blood pressure; DBP: diastolic blood pressure; FBG: fasting blood sugar; HbA1c: glycosylated hemoglobin; eGFR: estimated glomerular filtration rate; LDL-C: low-density lipoprotein cholesterol; HDL-C: high-density lipoprotein cholesterol; RAAS: renin–angiotensin–aldosterone system.

Data are presented as the mean ± standard, the median with interquartile range or counts and percentages.

A two-tailed *p* < 0.05 was considered statistically significant.

Furthermore, according to the cut off value of 10-year ASCVD risk of 7.5%, the total 218 patients were divided into two groups: group 1 (ASCVD risk score < 7.5%, *n* = 54) and group 2 (ASCVD risk score ≥ 7.5%, *n* = 164). Patients in group 2 had significant male predominance, older age, higher hemoglobin and LDL-C level, lower HDL-C level (Supplementary Table 2).

### Pathological characteristics

As listed in Table 2, baseline pathological features of all enrolled patients were stated. Referring to the glomerular classification of RPS criteria in 2010 [16], there were 8 patients (3.7%) in class I, 52 (23.9%) in class IIa, 22 (10.1%) in class IIb, 99 (45.4%) in class III, and 37 (17.0%) in class IV, respectively. Interstitial fibrosis and tubular atrophy (IFTA) score of 0, 1, 2 and 3 were observed in 4 (1.8%), 102 (46.8%), 88 (40.4%) and 24 (11.0%) patients. For interstitial inflammation, 11 (5.0%), 175 (80.3%) and 32 (14.7%) patients were scored as 0,1 and 2, respectively. For arteriolar hyalinosis, 23 (10.6%), 110 (50.7%) and 84 (38.7%) were scored as 0, 1 and 2, respectively. There were no significant differences in the distribution of glomerular classes, IFTA scores and severity of arteriolar hyalinosis between two groups, except interstitial inflammation score. The score of interstitial inflammation:0, 1 and 2, accounted for 2.8%, 74.3% and 22.9%, respectively, for patients with higher ASCVD risk score, and 7.3%, 86.2% and 6.4% for those with lower ASCVD risk score (*p* = 0.001). Baseline pathological characteristics of two groups divided by ASCVD risk score of 7.5% were presented in Supplementary Table 3, and there was significant difference in interstitial inflammation score rather than other three pathological lesion scores. Spearman correlation analysis showed that ASCVD risk score was positively associated with interstitial inflammation (*r* = 0.218, *p* = 0.001), while no significant association was observed between other pathological lesions and ASCVD risk score (Table 2).

**Table 2.** Baseline pathologic characteristics of enrolled patients, and correlation between ASCVD risk and pathologic parameters.

Characteristics	All (*n* = 218)	Correlation coefficient (*r*)	*p* Value	ASCVD ris*k* < 14.1% (*n* = 109)	ASCVD ris*k* ≥ 14.1% (*n* = 109)	*p* Value
Glomerular class						
I	8 (3.7%)	0.013	0.848	6 (5.5%)	2 (1.8%)	0.638
IIa	52 (23.9%)			27 (24.8%)	25 (22.9%)	
IIb	22 (10.1%)			10 (9.2%)	12 (11.0%)	
III	99 (45.4%)			47 (43.1%)	52 (47.7%)	
IV	37 (17.0%)			19 (17.4%)	18 (16.5%)	
IFTA						
0	4 (1.8%)	0.065	0.342	2 (1.8%)	2 (1.8%)	0.540
1	102 (46.8%)			55 (50.5%)	47 (43.1%)	
2	88 (40.4%)			39 (35.8%)	49 (45.0%)	
3	24 (11.0%)			13 (11.9%)	11 (10.1%)	
Interstitial inflammation						
0	11 (5.0%)	0.218	0.001	8 (7.3%)	3 (2.8%)	0.001
1	175 (80.3%)			94 (86.2%)	81 (74.3%)	
2	32 (14.7%)			7 (6.4%)	25 (22.9%)	
Arteriolar hyalinosis						
0	23 (10.6%)	–0.020	0.765	13 (12.0%)	10 (9.2%)	0.560
1	110 (50.7%)			51 (47.2%)	59 (54.1%)	
2	84 (38.7%)			44 (40.7%)	40 (36.7%)	

IFTA: interstitial fibrosis and tubular atrophy.

A two-tailed *p* < 0.05 was considered statistically significant.

### The ASCVD risk score and renal dysfunction

Univariate logistic regression analysis was performed to determine the risk factors for renal dysfunction among these patients. As Table 3 showed, DR, hemoglobin, uric acid, albumin, HbA1c, proteinuria and all four kinds of pathological lesions of DKD were associated with a higher risk of renal dysfunction. What’s more, ASCVD risk score level (≥14.1%) was also a risk factor in univariate logistic regression (OR, 2.288; 95% CI, 1.326–3.947; *p* = 0.003), and remained to be an independent risk factor after multivariable adjustments made for baseline clinical parameters and pathological scores (Model 1, OR, 3.561, 95% CI, 1.413–8.976, *p* = 0.007; Model 2, OR, 3.997, 95% CI, 1.385–11.530, *p* = 0.010).

**Table 3.** Univariate and multivariate logistic regression analysis of risk factors of renal dysfunction.

Variables	OR	95% CI	*p* Value
Univariate analysis			
Age (years)	1.033	0.997–1.071	0.074
Gender (male)	1.781	0.978–3.243	0.059
DR (yes)	1.769	1.023–3.059	0.041
Smoker (yes)	1.071	0.628–1.828	0.801
SBP (mmHg)	1.002	0.989–1.014	0.798
DBP (mmHg)	0.993	0.973–1.014	0.508
Hemoglobin (g/L)	0.961	0.948–0.974	<0.001
ASCVD risk score	1.016	0.996–1.036	0.116
ASCVD risk score ≥ 14.1%	2.288	1.326–3.947	0.003
FBG (mmol/L)	0.972	0.908–1.040	0.412
Uric acid (μmol/L)	1.007	1.003–1.011	<0.001
Serum albumin (g/L)	0.940	0.905–0.978	0.002
Triglyceride (mmol/L)	0.907	0.747–1.102	0.327
Total cholesterol (mmol/L)	1.038	0.811–1.329	0.767
LDL–C (mmol/L)	1.000	0.749–1.335	1.000
HDL–C (mmol/L)	1.406	0.736–2.687	0.302
HbA1c (%)	0.727	0.601–0.879	0.001
24–h proteinuria (g/d)	1.105	1.027–1.190	0.008
Glomerular class			
I + IIa			Reference
IIb	3.333	1.165–9.534	0.025
III	5.333	2.523–11.273	<0.001
IV	10.400	3.961–27.309	<0.001
IFTA			
0 + 1			Reference
2	4.234	2.316–7.739	<0.001
3	8.226	2.790–24.256	<0.001
Interstitial inflammation			
0 + 1			Reference
2	5.300	2.069–13.575	0.001
Arteriolar hyalinosis			
0			Reference
1	6.310	1.771–22.474	0.004
2	8.704	2.399–31.581	0.001
Model 1			
ASCVD risk score ≥ 14.1%	3.561	1.413–8.976	0.007
Model 2			
ASCVD risk score ≥ 14.1%	3.997	1.385–11.530	0.010

R: diabetic retinopathy; SBP: systolic blood pressure; DBP: diastolic blood pressure; ASCVD: atherosclerotic cardiovascular disease; FBG: fasting blood sugar; LDL-C: low-density lipoprotein cholesterol; HDL-C: high-density lipoprotein cholesterol; HbA1c: glycosylated hemoglobin; IFTA: interstitial fibrosis and tubular atrophy.

Model 1: Adjusted for diabetic retinopathy (DR) (yes or no), hemoglobin, uric acid, serum albumin, HbA1c, 24-h urine protein.

Model 2: Adjusted for variates in Model 1 and pathological lesions, including glomerular class, interstitial fibrosis and tubular atrophy score (IFTA), interstitial inflammation score and arteriolar hyalinosis.

A two-tailed *p* < 0.05 was considered statistically significant.

### The ASCVD risk score and renal outcomes

**Figure 2.**
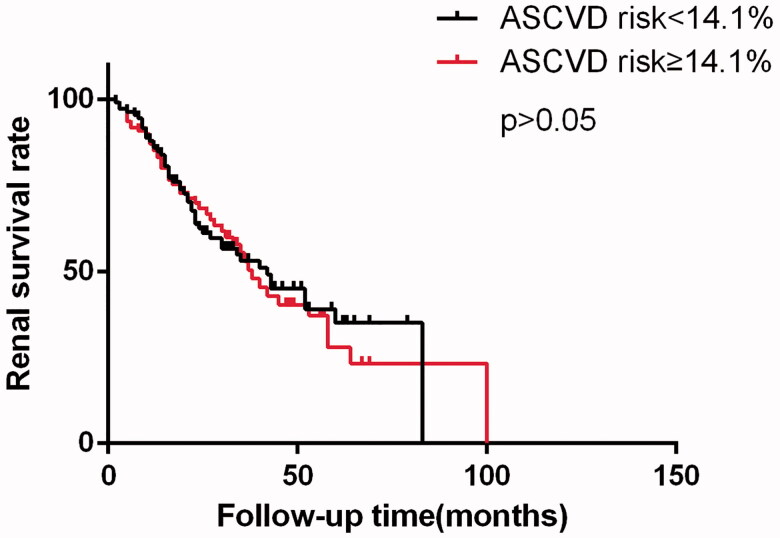
Kaplan–Meier curve of enrolled patients.

To determine whether high ASCVD risk score could predict renal outcome of diabetic nephropathy patients, the Kaplan–Meier survival analysis and Cox regression were performed. The 3- and 5- year renal survival rates were 53.0% and 35.1% for lower risk group, 52.9% and 27.9% for higher risk group, respectively. There was no significant difference in renal survival rate observed between two groups (Figure 2). In univariate and multivariate Cox regression analysis, higher ASCVD score (≥14.1%) was not a predictor of renal prognosis in our present cohort (Table 4).

**Table 4.** Univariate and multivariate Cox regression analysis of prognosis of enrolled patients.

Variables	Univariate			Multivariate		
	HR	95% CI	*p* Value	HR	95% CI	*p* Value
Age (years)	0.979	0.953–1.006	0.129	0.963	0.924–1.004	0.073
Gender (male)	1.351	0.855–2.136	0.198	1.386	0.687–2.800	0.362
DR (yes)	2.130	1.409–3.218	<0.001	1.741	1.064–2.849	0.027
SBP (mmHg)	1.003	0.994–1.013	0.478			
DBP (mmHg)	0.996	0.981–1.012	0.655			
Duration of diabetes (months)	1.002	0.999–1.005	0.166			
Smoker (yes)	1.180	0.790–1.763	0.420			
Hemoglobin (g/L）	0.966	0.958–0.975	<0.001	0.996	0.981–1.011	0.595
ASCVD risk score	0.994	0.979–1.010	0.485			
ASCVD risk score ≥ 7.5%	1.035	0.651–1.647	0.883			
ASCVD risk score ≥ 14.1%	1.031	0.69–1.539	0.883	0.928	0.478–1.802	0.825
eGFR (ml/min/1.73m^2^)	0.969	0.961–0.978	<0.001	0.970	0.957–0.982	<0.001
Uric acid (μmol/L)	0.999	0.997–1.002	0.579			
Serum albumin (g/L)	0.884	0.858–0.910	<0.001	0.911	0.870–0.955	<0.001
Triglyceride (mmol/L)	0.835	0.702–0.994	0.042	0.893	0.666–1.197	0.449
Total cholesterol (mmol/L)	1.157	0.963–1.391	0.120			
LDL-C (mmol/L)	1.154	0.924–1.442	0.207			
HDL-C (mmol/L)	1.786	1.133–2.815	0.012	1.340	0.736–2.438	0.339
HbA1c (%)	0.905	0.794–1.031	0.135			
24-h urine protein (g/d)	1.106	1.073–1.140	<0.001	1.017	0.958–1.079	0.587
Glomerular class I + IIa			<0.001			0.290
IIb	2.488	1.024–6.046	0.044	0.369	0.116–1.167	0.090
III	5.123	2.608–10.062	<0.001	0.829	0.343–2.008	0.687
IV	4.482	2.061–9.748	<0.001	0.742	0.258–2.133	0.579
IFTA 0 + 1			<0.001			0.129
2	2.379	1.525–3.711	<0.001	1.108	0.632–1.943	0.720
3	2.165	1.120–4.184	0.022	0.464	0.178–1.208	0.116
Interstitial inflammation score (2 vs. 0 + 1)	3.195	1.992–5.122	<0.001	1.647	0.900–3.014	0.106
Arteriolar hyalinosis 0			0.010			0.348
1	2.831	1.112–7.204	0.029	2.102	0.757–5.838	0.154
2	3.905	1.554–9.812	0.004	1.760	0.637–4.865	0.276

DR: diabetic retinopathy; SBP: systolic blood pressure; DBP: diastolic blood pressure; ASCVD: atherosclerotic cardiovascular disease; eGFR: estimated glomerular filtration rate; LDL-C: low-density lipoprotein cholesterol; HDL-C: high-density lipoprotein cholesterol; HbA1c: glycosylated hemoglobin; IFTA: interstitial fibrosis and tubular atrophy.

A two-tailed *p* < 0.05 was considered statistically significant.

## Discussion

In the present study, we enrolled 218 T2DM patients with biopsy-proven DKD and without known CVD history. 10-year ASCVD risk score was calculated and compared between different eGFR categories, which showed that about 75.2% patients with DKD were at high risk of 10-year ASCVD event and comparable risk score was found among patients in different stages. DKD patients, even in CKD stage 1, had comparable ASCVD risk score to patients in CKD stage 2, 3, and 4. Patients with higher risk (≥14.1%) for 10-year ASCVD event had an older age, higher SBP, more obvious male predominance, more smokers, higher triglyceride level, and lower HDL-C and eGFR level than those with lower risk. As for histological lesion, glomerular class, IFTA and arteriolar hyalinosis showed no statistical difference between two groups. However, interstitial inflammation was more severe in patients with higher ASCVD risk score than those with lower risk score. The renal survival rate was comparable between the two risk score groups.

ASCVD, a major kind of CVD, is the leading cause of morbidity and mortality worldwide [20]. In a prospective study of association between lipids and risk of ASCVD, higher triglyceride and lower HDL-C was associated with higher ASCVD event respectively [21], which was in accordance with our results. Smoking, DM, and hypertension were also regarded as risk factors of ASCVD in many studies [22–24]. Accumulated evidence suggested that CKD was an independent risk factor for ASCVD, and the interrelation becomes stronger in patients with declined eGFR [25,26]. Among the placebo group of SHARP study, the rate of 4-point major atherosclerotic cardiovascular events distinguished between eGFR level: 6.8% for CKD stages 1 and 2,10.4% for CKD 3,12.7% for CKD 4,13.3% for CKD 5 without dialysis and 16.5% for CKD 5 on dialysis, respectively [27]. Recent published European Society of Cardiology (ESC) and European Atherosclerosis Society (EAS) guidelines for dyslipidemia classified patients with CKD 3 as ‘high-risk population’ of 10-year coronary heart disease, and patients with CKD 4-5 as ‘very high-risk population’ [28]. Besides CKD, the coexistence of T2DM, known as ASCVD equivalent, increases the risk and mortality of ASCVD as well. According to cardiovascular and diabetes mellitus section of American Heart Association in 2015, the risk of dying from CVD is 2 to 4-fold of diabetic patients compared with non-diabetic patients [29]. The association between DKD and future ASCVD event hasn’t been well learned. Being different with general CKD patients, DKD patients in CKD stage 1 in our study were already at high risk of ASCVD with median ASCVD risk score of 10.9%, which should be classified as ‘high-risk population’.

The Pooled Cohort Equation, first published as a part of ACC/AHA guideline in 2013, has been well developed in estimating the future 10-year risk for ASCVD. PCE is subsequently validated in different populations and turned to have good discrimination and calibration [13,30]. Considering the poor CVD prognosis of patients with CKD, many studies were conducted to investigate the relation between PCE-estimated ASCVD risk and renal function. Tyson et al. [31] conducted a second analysis of Exercise and Nutritional Interventions for Cardiovascular Health (ENCORE) trial in participants with eGFR ≥ 60mL/min/1.73 m^2^. They found that the ASCVD risk (estimated using PCE) increased 2.7% with every decline of eGFR by 15 mL/min/1.73 m^2^.A Chinse epidemiological study enrolled 259657 patients to investigated the association of eGFR with 10-year ASCVD risk. Their data showed that patients with insufficient kidney function confronted higher 10-year ASCVD risk, compared with those with normal renal function [32]. However, the estimated 10-year ASCVD risk in DKD patients remained unclear. In our cohort, about 75.2% patients with DKD were at high risk of 10-year ASCVD event, and eGFR was negatively correlated with ASCVD risk, which was consistent with other studies on general chronic kidney diseases [31–33]. However, there seemed to be no significant difference in ASCVD risk score among different CKD stages. This could because diabetes and kidney dysfunction were both strong indicators of ASCVD, which led to high ASCVD risk of DKD patients even in CKD stage 1. Considering comparable 10-year ASCVD risk score between different eGFR stages, ASCVD prevention in DKD patients deserves greater attention even at early stage of DKD than general CKD population.

Ample studies have investigated the interaction between kidney and CVD, while most of them are focusing on impaired kidney function increasing CVD risk and mortality, such as ACCORD, ALLHAT [34,35]. However, the impact of CVD on renal function and possible mechanisms are rarely learned and less clear. Myocardial infarction (MI), a kind of ASCVD, is reported to cause renal function loss of about 3 mL/(min•year) to individuals with normal renal function [36]. In a rat model of CKD induced by unilateral nephrectomization, proteinuria and plasma creatinine increased in MI group significantly compared with control group. Renal interstitial damage and focal glomerulosclerosis were more severe in MI group than those in control group [37]. The number of macrophages in glomeruli was higher in MI group. A possible mechanism was related to the systemic or focal renal inflammation, which was derived from severe inflammation reaction in kidney after MI [37,38]. Traditional risk factors of ASCVD, such as hypertension, dyslipidemia, obesity, and metabolic syndromes can damage the kidney directly and by promoting intrarenal atherogenesis, even in the absence of obstructive lesions in the renal artery. Logistic analysis in our study also showed that the higher ASCVD risks core was associated with renal dysfunction. Although the estimated ASCVD risk score was negatively associated with baseline eGFR, the estimated ASCVD risk score was not an independent risk factor for progression to ESRD. Additionally, the presence of DR, lower serum albumin and eGFR were independently associated with renal outcomes. This inconsistency could be due to different characteristics of our patients, such as race, baseline eGFR and medical complications. Our participants had DM and were diagnosed with DKD by renal biopsy. Considering DM as a proinflammatory disease and strong indicator of ASCVD, the inflammation reaction induced by CVD could add little extra damage to kidney.

Diabetes, a disorder of glucose metabolism, can lead to macrovascular complications, which are similar to atherosclerotic lesion both in morphology and function, and microvascular complications (retinopathy, nephropathy, etc.) [39]. Proper glycemic control was supposed to release the heavy burden of diabetic complications, and several trials of glucose control in diabetic patients were conducted. ADVANCE and VADT indicated the benefits of intensive glucose control to microvascular endpoints, but no significant improvement of macrovascular outcomes [40,41]. Why glucose control had this paradoxical effect on diabetic micro- and macro-vascular complications remained obscure. There were structural and functional differences between macro- and microvessels. Macrovessels mainly provided blood to organs, while microvessels, worked as the smallest function unit of cardiovascular system, delivered nutrients to local tissue and took part in blood pressure maintenance [42]. For diabetic microangiopathy, vascular damage induced by intracellular hyperglycemia occurred early in the diabetes course and finally led to typical pathological changes in the vasculature. The most common vasculature change was thickened basement membrane, which could be due to overproduction of extracellular matrix proteins [43]. Several pathogenic theories of diabetic microvascular complications had been reported, including production of advanced glycation products, increased oxidative stress and reactive oxygen species, existence of low-grade inflammation and protein kinase C activation [43]. Macroangiopathy in diabetes was characterized by development of atherosclerosis. Several metabolic abnormalities, including hyperglycemia, insulin resistance and dyslipidemia, acted on different cells of macro-vasculature and platelets [44]. Besides those pathogenetic mechanisms mentioned above in diabetic microangiopathy, platelet and coagulation system activation was also involved in diabetic macroangiopathy. Increased clotting factors and plasminogen activator inhibitor-I, and decreased antithrombin III contribute to atherosclerosis.

The underlying mechanisms of differences between diabetic macro- and micro-vascular diseases were further investigated. As a antiangiogenic and proatherogenic protein, thrombospondin-1 (TSP-1) was reported to be involved in diabetic macro- and micro-vascular complications with different expression. TSP-1 was upregulated in large arteries while downregulated in microvascular epithelial cell of diabetic animals [45]. Endothelial cell played as a vital mediator in macro- and micro-vascular diseases. Endothelial cells arising from different vessels expressed different phenotypes under normal physiological conditions, and reacted differently in disease status [46]. Endothelial function measured by invasive flow-mediated vasodilatation (FMD) of brachial artery, was associated with microangiopathy, not macroangiopathy assessed by intimal–medial complex thickness (IMT) in patients with T2DM [47]. It could be the different pathogenic mechanism between micro- and macro-vascular complications, that explained non-predictable role of ASCVD risk for renal prognosis. More further investigations are needed.

There are several limitations in our study. First, this is a retrospective study, which means that we can’t exclude some other factors that may influence analysis results, such as life style, eating habits, etc. Second, there is inevitable bias of selecting participants, because of the indications of renal biopsy for diabetic patients. Our renal biopsies for diabetic patients were mainly performed for those with a rapid declining eGFR or suddenly increased proteinuria, especially those without DR and/or long DM duration. Third, based on the strict application of the Pooled Cohort Equation used in this study, patients younger than 40 years or older than 79 years were not included in this study. Included patients also had to meet the restricted range for blood pressure, cholesterol and HDL-C level, which leads to amounts of data loss of ASCVD risk. Finally, this study is completed in a single center, causing limited sample size and unanalyzed racial and geographical differences.

## Conclusions

In conclusion, our study demonstrated the negative correlation between eGFR and 10-year ASCVD risk in patients with biopsy-proven DN. However, the estimated 10-year ASCVD risk calculated by PCE failed to be an independently risk factor for progression to ESRD in patients with T2DM and DKD. More large-sample and prospective study is needed to explore and determine the interrelation between ASCVD risk and renal dysfunction.

## Supplementary Material

Supplemental Material

Supplemental Material

Supplemental Material
