# Supplementary material for: Association between atherosclerotic cardiovascular diseases risk and renal outcome in patients with type 2 diabetes mellitus
Source: Ren Fail. 2021 Mar 9;43(1):477–87. doi: 10.1080/0886022X.2021.1893186 (PMC7946063; doi:10.1080/0886022X.2021.1893186)
Supplement: Supplemental Material [file IRNF_A_1893186_SM5790.pdf]

Additional file 1: Characteristics of excluded and included patients.

Characteristics	Included patients (n=218)	Excluded patients (n=138)	P value
Gender(male,%)	155(71.1%)	96(69.6%)	0.757
Age(years)	52(48-58)	51(43-58)	0.065
DR(yes,%)	99(46.7%)	55(40.7%)	0.276
SBP(mmHg)	144.33±22.03	147.04±26.40	0.296
DBP(mmHg)	85.36±13.01	87.40±13.40	0.156
Duration of diabetes(months)	96(36-144)	84(36-120)	0.032
Smoker(yes,%)	104(47.7%)	62(45.6%)	0.698
Hemoglobin(g/L)	118.74±27.63	118.05±28.51	0.823
FBG(mmol/L)	7.40(5.51-9.56)	7.23(5.70-10.07)	0.953
HbA1c(%)	7.30(6.30-8.60)	7.20(6.20-8.20)	0.594
eGFR(ml/min/1.73m^2)	60.75(43.31-92.50)	56.58(39.67-91.40)	0.209
Serum creatinine(umol/L)	115.5(80.0-159.0)	126.5(88.0-160.0)	0.263
Uric acid(umol/L)	384.31±76.78	386.66±95.16	0.798
Serum albumin(g/L)	35.10(28.70-40.20)	31.75(25.20-38.90)	0.003
Triglyceride(mmol/L)	1.78(1.26-2.33)	1.69(1.28-2.42)	0.878
Total cholesterol(mmol/L)	5.00(4.35-5.74)	5.08(3.75-6.89)	0.606
LDL-C(mmol/L)	2.90(2.33-3.64)	2.94(1.96-4.24)	0.625
HDL-C(mmol/L)	1.21(1.02-1.53)	1.28(0.97-1.71)	0.926
24-h proteinuria(g/24h)	4.09(2.17-7.04)	5.16(2.48-8.70)	0.066
Pathological lesions			
Glomerular class			
Ⅰ	8(3.7%)	9(6.5%)	0.466
Ⅱa	52(23.9%)	28(20.3%)	
Ⅱb	22(10.1%)	12(8.7%)	
Ⅲ	99(45.4%)	71(51.4%)	
Ⅳ	37(17.0%)	18(13.0%)	
IFTA			
0	4(1.8%)	6(4.3%)	0.313
1	102(46.8%)	58(42.0%)	
2	88(40.4%)	53(38.4%)	
3	24(11.0%)	21(15.2%)	
Interstitial inflammation			

0	11(5.0%)	11(8.0%)	
1	175(80.3%)	97(70.3%)	0.096
2	32(14.7%)	30(21.7%)	
Arteriolar hyalinosis			
0	23(10.6%)	15(10.9%)	
1	110(50.7%)	70(50.7%)	0.996
2	84(38.7%)	53(38.4%)	
Therapy			
Insulin therapy(%)	154(70.6%)	102(73.9%)	0.503
Oral antidiabetic drugs(%)	99(45.4%)	59(42.8%)	0.623
RAAS inhibitor(%)	175(80.3%)	107(77.5%)	0.535
Lipid-lowering therapy(%)	132(60.6%)	82(59.4%)	0.832

Footnotes:

DR, diabetic retinopathy; SBP, systolic blood pressure; DBP, diastolic blood pressure; FBG, fasting blood sugar; HbA1c, glycosylated hemoglobin; eGFR, estimated glomerular filtration rate; LDL-C, low-density lipoprotein cholesterol; HDL-C, high-density lipoprotein cholesterol; IFTA, interstitial fibrosis and tubular atrophy; RAAS, renin-angiotensin-aldosterone system.

Data are presented as the mean ± standard, the median with interquartile range or counts and percentages.

A two-tailed $p < 0.05$ was considered statistically significant.
