# Supplementary material for: Association between atherosclerotic cardiovascular diseases risk and renal outcome in patients with type 2 diabetes mellitus
Source: Ren Fail. 2021 Mar 9;43(1):477–87. doi: 10.1080/0886022X.2021.1893186 (PMC7946063; doi:10.1080/0886022X.2021.1893186)
Supplement: Supplemental Material [file IRNF_A_1893186_SM5780.pdf]

Supplementary table 2. Baseline clinical characteristics of enrolled patient divided into two groups based on 7.5% of ASCVD risk score

| Characteristics | ASCVD risk score groups | | P value |
|---|---|---|---|
| | <7.5% （n=54） | ≥7.5% （n=164） | |
| Male,n( %） | 18(33.3) | 137(83.5) | <0.001 |
| Age(years) | 48(44-51) | 54(49-61) | <0.001 |
| DR(yes,%) | 26(48.1) | 73(46.2) | 0.805 |
| SBP(mmHg) | 139.46±20.88 | 145.93±22.22 | 0.061 |
| DBP(mmHg) | 87.17±15.89 | 84.77±11.92 | 0.311 |
| Duration of diabetes(months) | 78(36-132) | 108(36-156) | 0.269 |
| Smoker, n (%) | 2 (3.7) | 103(62.8) | <0.001 |
| Hemoglobin(g/L) | 108.80±23.85 | 121.94±28.08 | 0.002 |
| FBG(mmol/L) | 7.29(5.71-9.10) | 7.40(5.50-9.71) | 0.688 |
| HbA1c(%) | 7.10(6.10-8.45) | 7.40(6.40-8.60) | 0.397 |
| eGFR(>15ml/min/1.73m$^2$) | 60.75(45.21-92.71) | 60.87(42.96-91.51) | 0.636 |
| Serum creatinine(μmol /L) | 107.7(73.0-159.0) | 119.0(84.0-159.0) | 0.162 |
| Uric acid(μmol/L) | 379.70±79.02 | 385.82±76.21 | 0.612 |
| Serum albumin(g/L) | 36.00(29.40-41.40) | 35.00(28.40-40.00) | 0.601 |
| Triglyceride(mmol/L) | 1.71(1.28-2.15) | 1.81(1.26-2.42) | 0.659 |
| Total cholesterol(mmol/L) | 4.90(4.32-5.50) | 5.11(4.36-5.89) | 0.157 |
| LDL-C(mmol/L) | 2.58(2.28-3.45) | 2.99(2.42-3.69) | 0.035 |
| HDL-C(mmol/L) | 1.43(1.21-1.71) | 1.16(0.99-1.44) | <0.001 |
| 24-h proteinuria(g/d) | 3.74(2.24-5.95) | 4.28(2.17-7.77) | 0.269 |
| ASCVD risk score (%) | 4.2(2.4-6.0) | 19.7(12.1-31.8) | <0.001 |
| Therapy | | | |
| Insulin therapy(%) | 35(64.8%) | 119(72.6%) | 0.278 |
| Oral antidiabetic drugs(%) | 28(51.9%) | 71(43.3%) | 0.273 |

| | | | |
|---|---|---|---|
| RAAS inhibitor(%) | 46(85.2%) | 129(78.7%) | 0.296 |
| Lipid-lowering therapy(%) | 35(64.8%) | 97(59.1%) | 0.460 |

Footnotes:

ASCVD, atherosclerotic cardiovascular disease; DR, diabetic retinopathy; SBP, systolic blood pressure; DBP, diastolic blood pressure; FBG, fasting blood sugar; HbA1c, glycosylated hemoglobin; eGFR, estimated glomerular filtration rate; LDL-C, low-density lipoprotein cholesterol; HDL-C, high-density lipoprotein cholesterol; RAAS, renin-angiotensin-aldosterone system.

Data are presented as the mean ± standard, the median with interquartile range or counts and percentages.

A two-tailed $p < 0.05$ was considered statistically significant.
