# Supplementary material for: Association between atherosclerotic cardiovascular diseases risk and renal outcome in patients with type 2 diabetes mellitus
Source: Ren Fail. 2021 Mar 9;43(1):477–87. doi: 10.1080/0886022X.2021.1893186 (PMC7946063; doi:10.1080/0886022X.2021.1893186)
Supplement: Supplemental Material [file IRNF_A_1893186_SM5771.pdf]

Supplementary table 3. Baseline pathologic characteristics of enrolled patients divided into two groups based on 7.5% of ASCVD risk score

Characteristics	ASCVD risk<7.5% （n=54）	ASCVD risk≥7.5% （n=164）	P value
Glomerular class			
I	5(9.3%)	3(1.8%)	0.134
IIa	10(18.5%)	42(25.6%)	
IIb	5(9.3%)	17(10.4%)	
III	23(42.6%)	76(46.3%)	
IV	11(20.4%)	26(15.9%)	
IFTA			
0	1(1.9%)	3(1.8%)	0.928
1	25(46.3%)	77(47.0%)	
2	21(38.9%)	67(40.9%)	
3	7(13.0%)	17(10.4%)	
Interstitial inflammation			
0	5(9.3%)	6(3.7%)	0.026
1	46(85.2%)	129(78.7%)	
2	3(5.6%)	29(17.7%)	
Arteriolar hyalinosis			
0	8(15.1%)	15(9.1%)	0.330
1	23(43.4%)	87(53.0%)	
2	22(41.5%)	62(37.8%)	

Footnotes: IFTA, interstitial fibrosis and tubular atrophy.

A two-tailed $p < 0.05$ was considered statistically significant
